# First Japanese Experience of Robotic-Assisted Low Anterior Resection Using the da Vinci 5: Incorporating Force Feedback and Case Insights in Rectal Cancer Surgery

**DOI:** 10.70352/scrj.cr.26-0086

**Published:** 2026-04-08

**Authors:** Yuka Iwami, Hidekazu Takahashi, Kyoko Kobayashi, Masakatsu Paku, Satoshi Ishikawa, Kazuya Iwamoto, Shohei Takaichi, Tomofumi Ohashi, Yujiro Nakahara, Kohei Murakami, Tadafumi Asaoka, Takeshi Omori, Ichiro Takemasa

**Affiliations:** Department of Gastroenterological Surgery, Osaka International Medical and Science Center, Osaka Keisatsu Hospital, Osaka, Osaka, Japan

**Keywords:** rectal cancer, robot-assisted surgery, da Vinci 5, artificial intelligence–based surgical analysis, force feedback

## Abstract

**INTRODUCTION:**

Colorectal cancer outcomes depend not only on disease stage but also on surgical quality, particularly in locally advanced rectal cancer. Robot-assisted surgery offers advantages over laparoscopy but lacks haptic feedback. The da Vinci 5 Surgical System introduces Force Feedback (FFB) technology, which transmits kinesthetic sensations to the surgeon and quantifies forces, as well as Case Insights, an artificial intelligence–based intraoperative data platform. To the best of our knowledge, this report describes the first case of robot-assisted low anterior resection for rectal cancer performed in Japan using da Vinci 5.

**CASE PRESENTATION:**

A woman in her 50s presented with constipation and was diagnosed with rectal cancer (cT3N0M0). She underwent robot-assisted low anterior resection using da Vinci 5. Console time was 131 min with minimal blood loss. The patient recovered uneventfully without leakage or urinary dysfunction and was discharged on POD 7. Case Insights revealed an instrument active time of 82% and average FFB forces of 2.1–2.7 N, with forces >6.5 N applied during only 4.3%–6.4% of the procedure, mainly during rectal mobilization. A complete total mesorectal excision and negative circumferential resection margins were achieved.

**CONCLUSIONS:**

Surgical skills have long remained tacit expert knowledge. FFB and Case Insights provide numeric metrics synchronized with intraoperative procedures, which may help convert tacit skills into explicit, quantifiable information. This enables experts to better understand their operative technique and help novices learn through objective, verbalized information that facilitates procedural understanding.

## Abbreviations

AI
: artificial intelligence
CRM
: circumferential resection margin
dV5
: da Vinci 5
FFB
: force feedback
TME
: total mesorectal excision

## INTRODUCTION

Colorectal cancer remains one of the most common malignancies worldwide,^1,2)^ and surgical quality plays a decisive role in determining both oncologic and functional outcomes. In locally advanced rectal cancer, TME has become the standard surgical principle, significantly reducing local recurrence.^3)^ Currently, distant metastasis represents the dominant recurrence pattern.^4)^ In addition to achieving a complete TME, securing a CRM is essential for long-term oncologic outcomes.^5)^ Furthermore, precise dissection along the mesorectal fascia while meticulously preserving the autonomic nerves within the narrow pelvic cavity is critical to minimize postoperative urinary and sexual function. These technical requirements underscore the importance of precise tissue handling and appropriate traction during rectal surgery.

Minimally invasive surgery has rapidly evolved in recent decades. Laparoscopic surgery has demonstrated comparable oncologic outcomes to open surgery^6)^ while offering improved short-term recovery.^7)^ However, the use of rigid instruments with limited degrees of freedom may restrict fine manipulation in the confined pelvic space. Robot-assisted surgery was introduced to overcome these technical limitations through the use of articulated instruments, stable 3D visualization, tremor filtration, and motion scaling. Large clinical trials have shown comparable or, in selected settings, improved surgical quality with robotic approaches, particularly regarding TME completeness and CRM negativity.^8–11)^ Nevertheless, conventional robotic surgical systems lack haptic sensation, requiring surgeons to rely exclusively on visual cues to estimate tissue tension and applied force. Although experienced surgeons can partially compensate for the absence of haptic feedback using visual information, haptic sensation is a fundamental component of human perception, and its integration into robot-assisted surgery has the potential to further improve surgical quality.

Haptic perception consists of 2 components: kinesthetic (force) and tactile (cutaneous) feedback. While tactile feedback remains technically challenging to implement in surgical systems, kinesthetic feedback can be reproduced by transmitting resistance to the surgeon’s hand controls. The absence of haptic sensation in robotic surgery has long been recognized as a potential limitation, particularly in procedures such as rectal surgery where excessive traction may lead to tissue injury or nerve damage. Experienced surgeons may partially compensate through visual assessment and accumulated tacit knowledge; however, the ability to objectively measure and reproduce optimal traction forces has remained limited.

The dV5 Surgical System (Intuitive Surgical, Sunnyvale, CA, USA) introduces 2 major technological innovations. First, its FFB technology provides real-time kinesthetic feedback to the surgeon while simultaneously quantifying the forces applied at the instrument tip. These force data are recorded as time-series information and can be analyzed postoperatively. Second, the system incorporates Case Insights, an AI–based intraoperative analysis platform that automatically integrates surgical video, instrument activity metrics, and force measurements. By synchronizing these data streams, Case Insights enables objective evaluation of console time, instrument usage, and force application patterns during specific operative phases.

The ability to quantify instrument forces represents a conceptual shift in surgical practice. Surgical skill has traditionally been regarded as tacit knowledge, transmitted through observation and apprenticeship rather than through measurable parameters. Quantitative visualization of applied forces may allow expert techniques to be analyzed, standardized, and potentially translated into objective educational benchmarks. In addition, excessive force application could theoretically be identified and correlated with intraoperative events or postoperative outcomes in future studies.

To the best of our knowledge, this report describes the first Japanese case of robot-assisted low anterior resection for rectal cancer performed using the dV5 system. We present not only the clinical course of this patient but also quantitative intraoperative analyses derived from FFB and Case Insights, highlighting their potential implications for surgical education and quality assessment.

## CASE PRESENTATION

A woman in her 50s with a BMI of 25.1 kg/m^2^ presented with constipation. Colonoscopy revealed a 35-mm type 2 lesion on the posterior rectal wall 7 cm from the anal verge. Biopsy revealed well-differentiated adenocarcinoma. MRI demonstrated a semi-circumferential cT3 lesion with a minimum CRM of 3 mm at the posterior wall. CT revealed no distant metastases or enlarged lymph nodes. Her history included robot-assisted right hemicolectomy for ascending colon cancer 2 years earlier, without recurrence. Comorbidities included mild hypertension and diabetes, with a hemoglobin A1c of 6.9%.

**Fig. 1:**
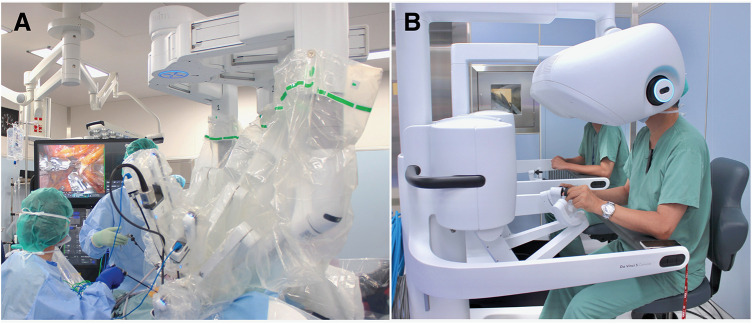
Operating room setup with the da Vinci 5 Surgical System (dV5). (**A**) In dV5, the components are renamed as follows: the patient cart is referred to as the cart, vision cart as the tower, and surgeon console as the console. (**B**) Dual console of dV5. Improved console ergonomics enhance the precision and usability of instrument control.

**Fig. 2:**
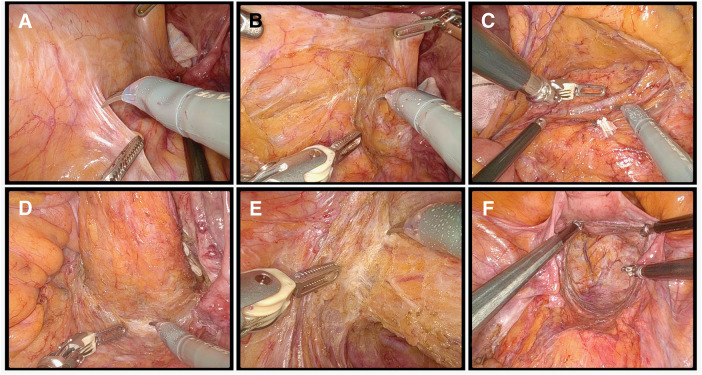
Intraoperative view using the da Vinci 5 Surgical System (dV5). (**A**) Dissection begins with a medial approach. (**B**) Dissection proceeds along the mesorectal fascia while preserving autonomic nerves. (**C**) D3 lymph node dissection is performed following division of the inferior mesenteric artery root. (**D**) Dissection of the dorsal side of the rectum. (**E**) Dissection of the left side of the rectum. (**F**) Pelvic nerves are preserved after rectal transection.

After the diagnosis of rectal cancer as cT3N0M0, clinical stage II, the patient underwent robot-assisted low anterior resection using dV5, performed by an experienced surgeon who had completed more than 1000 robot-assisted colorectal procedures in July 2025 (**Fig. 1**). Following a 4-cm umbilical incision, adhesions among the parietal peritoneum, small bowel, and greater omentum, probably caused by prior surgery, were identified and lysed. The peritoneal cavity was then inspected, and revealed no liver or peritoneal metastases. The patient was subsequently placed in a 20° head-down and 15° left-tilt position. The ports were placed as follows: an 8-mm port at the umbilical incision for arm 2, an 8-mm port in the lower right abdomen for arm 4, a 12-mm port between arms 2 and 4 for arm 3, and an 8-mm port in the upper left abdomen for arm 1. In addition, two 5-mm assistant ports were placed in the upper right and left abdomen. The small bowel was displaced to the upper right side of the abdomen to secure an adequate working space in the pelvis. Docking was performed on the left side by targeting the sigmoid–descending junction. Arm assignments were as follows: Arm 1 for FFB fenestrated bipolar; Arm 2 for endoscope; Arm 3 for monopolar curved scissors/FFB Maryland bipolar; and Arm 4 for FFB Cadiere. FFB was set to “low” among low, medium, and high FFB sensitivity levels. TME was initiated using the medial approach. The peritoneum was incised on the right side (**Fig. 2A**), and dissection proceeded cranially along the aorta, preserving the hypogastric and pelvic nerves (**Fig. 2B**). The ureter, ovarian artery, and veins were preserved in the retroperitoneum. The inferior mesenteric artery was divided with D3 lymph node dissection (**Fig. 2C**), followed by dissection of the inferior mesenteric vein and left colic artery. The peritoneal reflection was then incised, followed by mobilization of the descending colon and pelvic dissection posteriorly, laterally, and anteriorly to the pelvic floor (**Fig. 2D** and **2E**). The rectum was transected 3 cm distal to the tumor (**Fig. 2F**). The dV5 was undocked, and the colorectum was exteriorized through the umbilical incision. The proximal colon was transected 10 cm proximal to the tumor, and the specimen was removed. An indocyanine green perfusion assessment confirmed adequate blood flow at both ends of the planned anastomosis. Double-stapling technique anastomosis was then performed. The results of the air leak test were negative, and the wounds were closed without intraperitoneal drains.

**Fig. 3:**
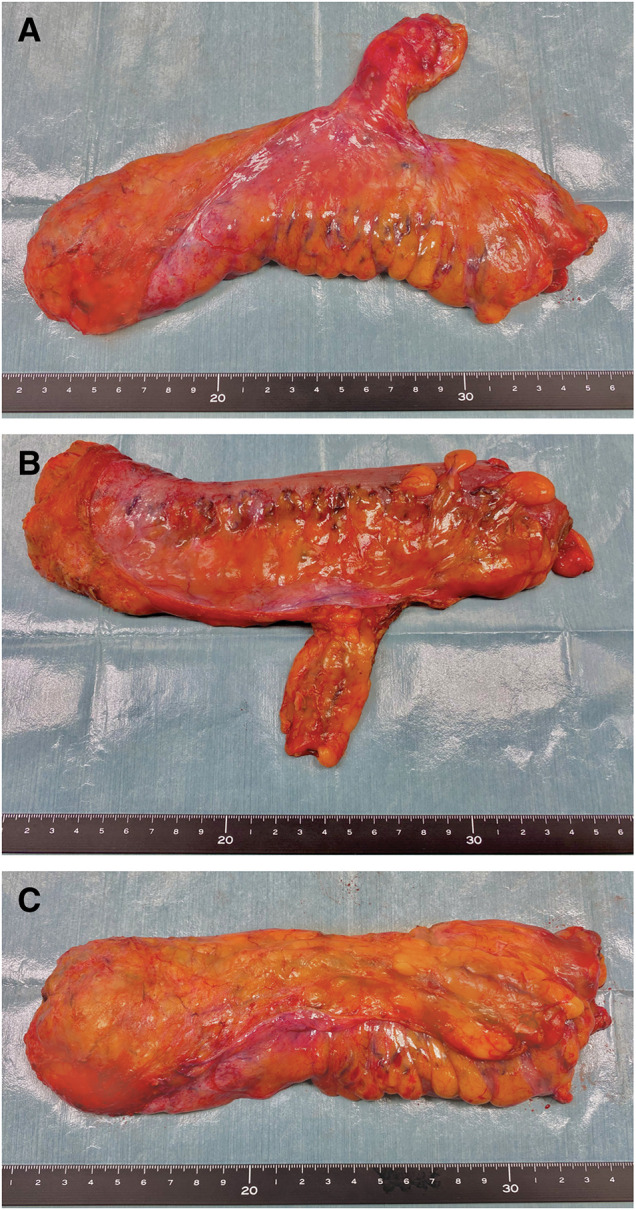
Resected specimen following complete total mesorectal excision. The mesorectal surface is smooth without defects. (**A**) Right lateral view of the resected specimen. (**B**) Left lateral view of the resected specimen. (**C**) Dissected plane of the resected specimen.

The console time was 131 min, with minimal blood loss. The patient recovered uneventfully without leakage or urinary dysfunction and was discharged on POD 7. Pathology revealed pT2N1aM0, stage IIIa, with a complete TME and negative margins (distal margin, 30 mm; proximal margin, 120 mm; CRM, 3 mm) (**Fig. 3**).

### Data from Case Insights

**Fig. 4:**
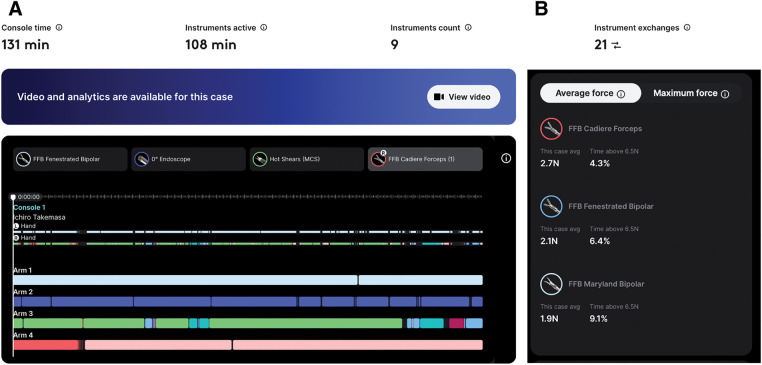
Case Insights data. Case Insights automatically records console time, instrument usage, stapling, and clipping events. (**A**) Time-series information for instrument usage for each robotic arm. (**B**) Average forces and time above 6.5 N are shown for each instrument. FFB, Force Feedback; MCS, Monopolar Curved Scissors; N, newtons

**Fig. 5:**
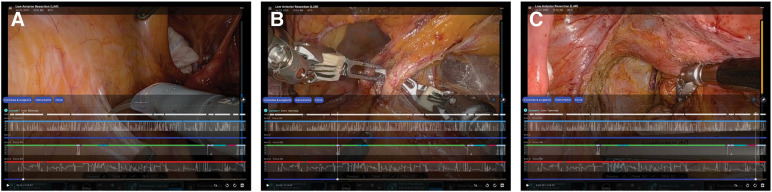
Case Insights screen. (**A**) At the beginning of the peritoneal incision. (**B**) At the division of the inferior mesenteric artery root. (**C**) During rectal mobilization. Higher forces were predominantly generated at this stage, particularly in Arm 4.

The endoscope and instrument information, including docking events and usage metrics, were automatically recorded in Case Insights (**Fig. 4**). The data showed a total console time of 131 min and an instrument activation time of 108 min (82%). Nine instruments were used during this procedure. For each instrument, the installation time, number of installations, and time of use were recorded. Twenty-one instrument exchanges were performed. Single-staple firing was performed, and 5 clips were applied using 2 different clip appliers. The forces applied to each instrument are reported in newtons (N) within a range of 0–6.5 N. Surgical videos are automatically stored online and can be accessed through the surgeon’s account. They were synchronized with intraoperative data, including instrument activities and force data, as shown in the line graphs (**Fig. 5**). In this study, 3 types of FFB instruments were used with the following parameters (**Fig. 4**):

• FFB Cadiere forceps (arm 4): mean 2.7 N, time in use 102 min, >6.5 N for 4.3% of time

• FFB Fenestrated Bipolar (arm 1): mean 2.1 N, time in use 95 min, >6.5 N for 6.4% of time

• FFB Maryland Bipolar (arm 3): mean 1.9 N, time in use 10 min, >6.5 N for 9.1% of time

In addition, the endoscope clutch was activated 834 times, and the energy pedal was used 988 times. Currently, clutch counts reflect only activation of the hand controller and do not include pedal controller inputs.

## DISCUSSION

To the best of our knowledge, this is the first Japanese case of robot-assisted low anterior resection using the dV5. The 2 key innovations are the FFB technology and AI-based Case Insights platform. With FFB, instrument forces are automatically captured as time-series data, enabling objective assessment of their magnitude and temporal patterns throughout the procedure. This allows kinesthetic information, previously appreciated only empirically as tacit knowledge, to be converted into visualized quantitative data. In robot-assisted rectal surgery, Arm 1 is essential for countertraction during triangulation, and Arm 4 plays a crucial role in exposure. However, full utilization of Arm 4 is challenging, and it has been reported that only surgeons proficient in robot-assisted surgery can optimally exploit Arm 4 through continuous adjustments.^12)^ In the present case, we used FFB Cadiere forceps on Arm 4 and FFB Fenestrated Bipolar on Arm 1. Average forces applied to these instruments ranged from 2.1 to 2.7 N, with forces exceeding the maximum measurement range of 6.5 N for 4.3%–6.4% of the time during which force was applied (**Fig. 4**). The clinical significance of the absolute force values measured in this study remains unclear. However, the achievement of a complete TME and negative CRM without intraoperative tissue injury, significant blood loss, or postoperative urinary dysfunction suggests that traction forces were appropriately controlled throughout the procedure. It is currently not possible to numerically quantify and analyze instrument forces by surgical phase because autosegmentation is not yet available for colorectal procedures in dV5. Nevertheless, qualitative review of the surgical video and FFB data indicated that higher forces were predominantly generated during rectal mobilization (**Fig. 5C**). This observation is consistent with a report by Chang et al., which demonstrated that abdominoperineal resection is associated with higher instrument forces than those of colon surgery.^13)^ This finding is also consistent with expert tacit knowledge that relatively stronger traction is required for rectal mobilization than for other phases of rectal surgery. These results suggest the potential of FFB to visualize and quantify tacit knowledge. This is particularly important because tacit knowledge, such as surgical skills and expert judgment, is inherently difficult to verbalize. By helping transform tacit knowledge into explicit, quantifiable information, FFB not only enables a more detailed understanding of surgical performance but also supports skill acquisition by novice surgeons.

Case Insights records console time, instrument usage, stapling, and clipping events and automatically synchronizes these data with surgical videos. This fully automated process not only reduces the time required for surgeons to collect data for analysis but also has the potential to improve overall operating room efficiency. In the present case, instrument active time was 108 min of 131 min of console time, indicating that instruments were active for 82% of console time and suggesting efficient console utilization. Synchronized visualization of FFB data with surgical videos enables objective mechanical characterization of each maneuver. Current specifications are limited to comparisons within a single surgeon, and AI functions are limited to segmentation for a limited number of procedures. However, integration of Case Insights functions with FFB data in rectal surgery may enable comparisons among multiple surgeons within the same surgical phase, which could help define optimal traction forces and safe ranges for each phase. Ultimately, this approach may enable standardized, data-driven performance benchmarks for education and quality improvement and further facilitate the conversion of tacit knowledge into explicit knowledge.

This study had several limitations. This was a single case performed by a highly experienced robotic colorectal surgeon, limiting its generalizability to broader practice and to surgeons early in the learning curve. Presently, the AI functions implemented in dV5 are mainly limited to segmentation, and their clinical application is restricted to a few procedures, including cholecystectomy, hernia repair, prostatectomy, and hysterectomy. Moreover, the clinical significance of forces exceeding 6.5 N remains unknown. In dV5, 6.5 N represents the upper limit of the device-defined reliable measurement range rather than a validated safety threshold. Tissue tolerance likely varies according to organ type and patient characteristics. Nakashima et al. reported that stronger grasping forces (3 N vs. 1 N) increased tissue damage in lung and liver, whereas gastrointestinal tissues were not damaged under forces up to 3 N.^14)^ Although derived from a different feedback system, these findings provide preliminary context for interpreting force data. Early clinical use of dV5 has also suggested that higher FFB sensitivity reduces time spent above 6.5 N.^15)^ However, safe force thresholds remain undefined, and future outcome-based studies are needed to establish organ-specific optimal ranges.

Future multicenter analyses are required to determine acceptable force thresholds and to correlate these thresholds with postoperative complications. Quantifying the optimal forces for critical steps, such as anterior rectal dissection, may help establish best practices. Ultimately, experienced surgeons may be able to reinterpret their techniques quantitatively, whereas novices can learn using objective metrics, potentially shortening the learning curve and enhancing surgical education.

## CONCLUSIONS

Robot-assisted low anterior resection of rectal cancer using dV5 was safely performed. In the future, FFB technology and AI-based Case Insights platforms are expected to advance further and help surgeons provide higher-quality surgery.
